# Potential common mechanism of four Chinese patent medicines recommended by diagnosis and treatment protocol for COVID-19 in medical observation period

**DOI:** 10.3389/fmed.2022.874611

**Published:** 2022-10-26

**Authors:** Lin Wang, Zheyi Wang, Zhihua Yang, Xingwang Wang, Liping Yan, Jianxiong Wu, Yue Liu, Baohui Fu, Hongtao Yang

**Affiliations:** ^1^Department of Nephrology, First Teaching Hospital of Tianjin University of Traditional Chinese Medicine, National Clinical Research Center for Chinese Medicine Acupuncture and Moxibustion, Tianjin, China; ^2^Qilu Hospital, Shandong University, Shandong, China; ^3^Institute of Traditional Chinese Medicine, Tianjin University of Traditional Chinese Medicine, Tianjin, China; ^4^College of Animal Science and National Engineering Research Center for Breeding Swine Industry, South China Agricultural University, Guangzhou, Guangdong, China; ^5^School of Pharmacy, Jiangxi University of Traditional Chinese Medicine, Nanchang, China

**Keywords:** COVID-19, Chinese patent medicines, active components, common mechanism of action, network pharmacology, ACE2, 3CL pro

## Abstract

The global epidemic has been controlled to some extent, while sporadic outbreaks still occur in some places. It is essential to summarize the successful experience and promote the development of new drugs. This study aimed to explore the common mechanism of action of the four Chinese patent medicine (CPMs) recommended in the *Medical Observation Period COVID-19 Diagnostic and Treatment Protocol* and to accelerate the new drug development process. Firstly, the active ingredients and targets of the four CPMs were obtained by the Chinese medicine composition database (TCMSP, TCMID) and related literature, and the common action targets of the four TCMs were sorted out. Secondly, the targets of COVID-19 were obtained through the gene-disease database (GeneCards, NCBI). Then the Venn diagram was used to intersect the common drug targets with the disease targets. And GO and KEGG pathway functional enrichment analysis was performed on the intersected targets with the help of the R package. Finally, the results were further validated by molecular docking and molecular dynamics analysis. As a result, a total of 101 common active ingredients and 21 key active ingredients of four CPMs were obtained, including quercetin, luteolin, acacetin, kaempferol, baicalein, naringenin, artemisinin, aloe-emodin, which might be medicinal substances for the treatment of COVID-19. TNF, IL6, IL1B, CXCL8, CCL2, IL2, IL4, ICAM1, IFNG, and IL10 has been predicted as key targets. 397 GO biological functions and 166 KEGG signaling pathways were obtained. The former was mainly enriched in regulating apoptosis, inflammatory response, and T cell activation. The latter, with 92 entries related to COVID-19, was mainly enriched to signaling pathways such as Coronavirus disease—COVID-19, Cytokine-cytokine receptor interaction, IL-17 signaling pathway, and Toll-like receptor signaling pathway. Molecular docking results showed that 19/21 of key active ingredients exhibited strong binding activity to recognized COVID-19-related targets (3CL of SARS-CoV-2, ACE2, and S protein), even better than one of these four antiviral drugs. Among them, shinflavanone had better affinity to 3CL, ACE2, and S protein of SARS-CoV-2 than these four antiviral drugs. In summary, the four CPMs may play a role in the treatment of COVID-19 by binding flavonoids such as quercetin, luteolin, and acacetin to target proteins such as ACE2, 3CLpro, and S protein and acting on TNF, IL6, IL1B, CXCL8, and other targets to participate in broad-spectrum antiviral, immunomodulatory and inflammatory responses.

## Introduction

Since the beginning of December 2019, COVID-19 has caused devastating consequences (1) and has been declared as the sixth public health emergency of international concern by the World Health Organization (WHO) (2). COVID-19 is highly contagious and spreads quickly, with broader impacts, and the population is generally susceptible (3). In addition, SARS-CoV-2 directly attacks the respiratory system and attacks the respiratory system, but also affects the immune, digestive, cardiovascular, neurological, genitourinary, and other extrapulmonary systems (4, 5). Moreover, related literature reports suggested that patients with COVID-19 might have multiple systemic sequelae during recovery (6). At present, the global epidemic has been controlled to some extent, while sporadic outbreaks still occur in some places. It is essential to summarize the successful experiences of various countries and promote the development of new drugs.

As one of the first countries affected by COVID-19, China has accumulated a lot of valuable experience in fighting COVID-19. It has been shown that Lianhua Qingwen (LHQW), Huoxiang Zhengqi (HXZQ), Jinhua Qinggan (JHQG), and Shufeng Jiedu (SFJD) are effective in treating COVID-19 (7). For COVID-19 patients in the medical observation period, JHQG, LHQW, and SFJD are recommended for fever and fatigue. For fatigue with gastrointestinal discomfort, HXZQ is recommended. In terms of clinical efficacy, in a multi-center randomized controlled study led by academician Nanshan Zhong, a total of 284 patients with COVID-19 were included. The results proved that combining LHQW with conventional treatment can significantly increase the symptom recovery rate, shorten the median time for symptom recovery, and improve the CT and clinical cure rates (8). Ling Chen et al. reviewed the clinical data of 200 patients with ordinary COVID-19 treated at Wuhan Third Hospital from January 27, 2020, to March 5, 2020. The results show that the combined application of SFJD can significantly improve the clinical symptoms of typical COVID-19 patients with cough, sputum, fatigue, chest tightness, and wheezing. The main symptoms are improved. It also can regulate the expression of related peripheral blood inflammation indicators and promote lungs inflammation absorption. At last, the cure rate are improved (9). Academician Boli Zhang and others reviewed 123 patients with mild COVID-19 who were admitted to the emergency department of *Hubei Integrated Traditional Chinese and Western Medicine Hospital* from February 1 to 5, 2020. The results demonstrated that JHQG combined with conventional western medicine treatment could significantly reduce the clinical symptoms of fever, cough, fatigue, and sputum in patients with mild COVID-19 and relieve anxiety of patients (10). HXZQ is an ancient anti-epidemic prescription. The Diagnosis and Treatment Scheme of Severe Acute Respiratory Syndrome (SARS) (Version 2004) recommends that HXZQ can be used for advanced lung closure, and it has shown initial results in the front line of anti-epidemic (11, 12).

Regarding basic experiments, corresponding evidence can be found for the treatment of COVID-19 by four CPMs. LHQW can work by inhibiting virus replication and reducing the release of cytokines from host cells. The team of academician Nanshan Zhong found in the African green monkey kidney cells Vero E6 cells that LHQW can inhibit the replication of SARS-CoV-2, affect the morphology of the virus, and inhibit pro-inflammatory cytokines at the mRNA level, such as TNF-α, IL-6, CCL2/MCP-1, and CXCL-10/IP-10 (13). SFJD has a broad spectrum of antiviral, anti-inflammatory, and antibacterial effects and can reduce lung injury (14), the levels of IL-1β and IL-18 in serum and bronchoalveolar perfusion fluid, and the expression levels of NLRP3-related components and virus titers in lung fluid (15–17). HXZQ has the functions of antiviral, anti-inflammatory, and immune regulation and could improve gastrointestinal discomfort (18). It can also reduce the inflammatory response in patients with COVID-19 by reducing pro-inflammatory factors, increasing the level of IL-10, and regulating the NF-kB pathway of inflammation (18). JHQG reduces lung inflammation, protects immune organs, regulates cytokine balance, and improves body immunity (19, 20).

**FIGURE 1:**
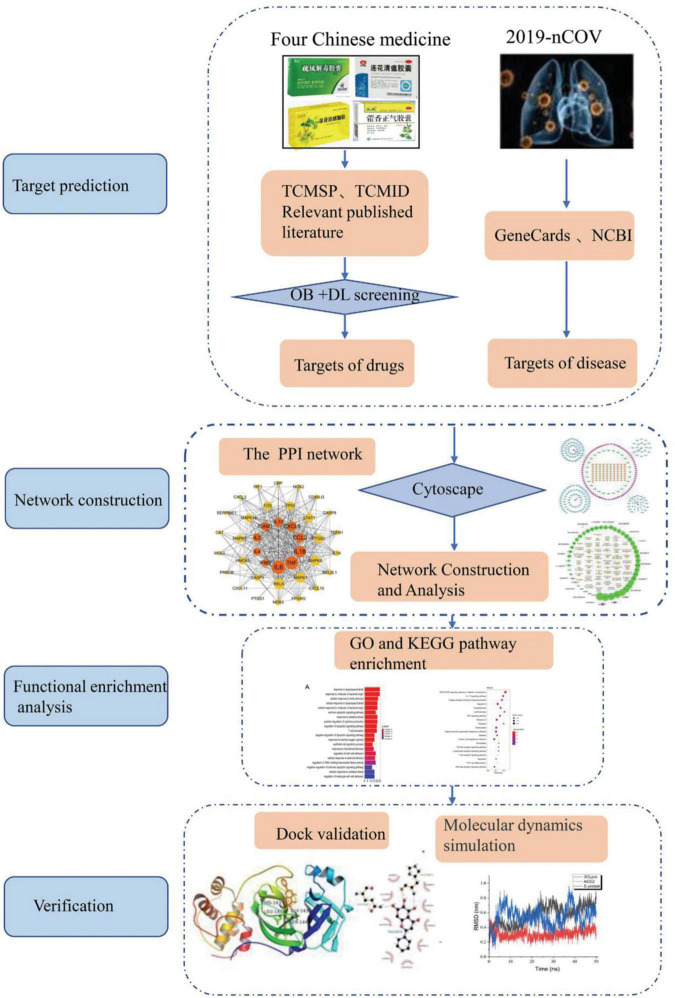
The workflow of this whole analysis for this study.

Evidence can be discovered in clinical trials and basic experiments for four CPMs to treat COVID-19. Is there a common mechanism of action among them? To promote the development of new drugs and provide a reference for the following basic experiment, we explored the possible mechanism of the action of four CPMs in the treatment of COVID-19 from the perspective of network pharmacology. The workflow is shown in Figure 1.

## Materials and methods

### Identification of candidate compounds

There were four prescriptions, one from each group. According to the composition of the prescriptions, the traditional Chinese medicine system (TCMSP)^1^ (21) database was searched for the components of the four prescriptions using the pinyin name or latin name of each herb in each prescription. The composition and dosage of each prescription are shown in Table 1. The herb not found in the TCMSP were supplemented from the TCMID^2^ database and relevant published literature. The TCMSP database is the most commonly used database for the retrieval of Chinese medicine ingredients and describes the relationships between drugs, targets, and diseases (21, 22). This database includes 500 TCMs from the 2010 edition of the pharmacopeia and 3,069 compounds (23).

**TABLE 1:** Herb composition of the four prescriptions.

Prescription name	Latin name/Kewscience website accepted name	Chinese pinyin name	Amount (%)
HXZQ	*Pogostemon cablin* (Blanco) Benth.	Guanghuoxiang	90 g (15.4%)
	*Atractylodes macrocephala* Koidz.	Baizhu	60 g (10.3%)
	*Magnolia officinalis* Rehd. et Wils.	Houpo	60 g (10.3%)
	*Pinellia ternata*	Banxia	60 g (10.3%)
	*Perilla frutescens* (L.) Britt.	zisu	30 g (5.1%)
	*Angelica dahurica* (Fisch. ex Hoffm.)Benth. et Hook. f. ex Franch. et Sav)	Baizhi	30 g (5.1%)
	*Citrus reticulata* Blanco	Chenpi	60 g (10.3%)
	*Poria cocos* (Schw.) Wolf	Fuling	30 g (5.10%)
	*Platycodon grandiflorus* (Jacq.) A. DC.	Jiegeng	60 g (10.3%)
	*Glycyrrhiza uralensis* Fisch.	Gancao	75 g (12.8%)
	*Areca catechu* L.	Dafupi	30 g (5.10%)
	*Ziziphus jujuba* Mill.	Dazao	A jujube
	*Zingiber officinale* Roscoe	Shengjiang	Three slices
JHQG	*Lonicera japonica* Thunb	Jinyinhua	
	*Ephedra sinica* Stapf	Mahuang	
	*Gypsum Fibrosuum*	Shigao	
	*Prunus armeniaca* L	Kuxingren	
	*Scutellaria baicalensis* Georgi	Huangqin	
	*Forsythia suspensa* (Thunb.) Vahl	Lianqiao	
	*Fritillaria thunbergii*	Zhebeimu	
	*Anemarrhena asphodeloides* Bunge	Zhimu	
	*Arctium lappa* L.	Niubangzi	
	*Artemisia carvifolia* Buch.-Ham. ex Roxb. Hort. Beng.)	Qinhao	
	*Mentha haplocalyx* Briq.	Bohe	
	*Glycyrrhiza uralensis* Fisch.	Gancao	
LHQW	*Forsythia suspensa* (Thunb.) Vahl	Lianqiao	
	*Lonicera japonica* Thunb	Jinyinhua	
	*Ephedra sinica* Stapf	Mahuang	
	*Prunus armeniaca* L	Kuxingren	
	*Gypsum Fibrosuum*	Shigao	
	*Isatis tinctoria*	Banlangeng	
	*Cyrtomium fortunei* J. Sm.	Guanzhong	
	*Houttuynia cordata* Thunb.	Yuxingcao	
	*Pogostemon cablin* (Blanco) Benth.	Guanghuoxiang	
	*Rheum palmatum* L.	Dahuang	
	*Rhodiola rosea* L.	Hongjingtian	
	*Mentha haplocalyx* Briq.	Bohe	
	*Glycyrrhiza uralensis* Fisch.	Gancao	
SFJD	*Isatis tinctoria*	Banlangeng	
	*Reynoutria japonica* Houtt.	Huzhang	
	*Forsythia suspensa* (Thunb.) Vahl	Lianqiao	
	*Phragmites communis* Trin.	Lugeng	
	*Patrinia scabiosifolia* Fisch. ex Trevir.	Baijiangcao	
	*Verbena officinalis* L.	Mabiancao	
	*Bupleurum chinense* DC	Chaihu	
	*Glycyrrhiza uralensis* Fisch.	Gancao	

### ADME screening

Oral bioavailability (OB) refers to the percentage of unmodified drug that is absorbed into the circulatory system after oral administration. The OB is a reliable indicator of the efficacy of oral administration (24). The higher the OB value of the drug is, the greater the clinical value is. After calculation and prediction by of the OBioavail1.1 software, herb components with an OB ≥ 30% were selected as candidate molecules for further analysis in this study (25).

The drug-likeness (DL) refers to the similarity of a compound to a known drug (26). The valuation of the DL coefficient is used in the early stages of drug development to help identify excellent compounds. Therefore, in this study, the tanimoto coefficient (27) was used to evaluate the DL index of the molecule of the four prescriptions. The calculation formula is as follows:


$$T\left(a,b\right)=\frac{a^{*}b}{a^{2}+b^{2}-ab}$$

where “a” is the molecular property of the herbal compound on the basis of Dragon software^3^ (28) and “b” is the average molecular property of all the molecules in the DrugBank database.^4^ The average DL index of all the drugs in this database is 0.18. Therefore, compounds that met the conditions of OB ≥ 30% and DL ≥ 0.18 were defined as active ingredients in this study and were considered to have better pharmacologic effects (29, 30).

### Identification of drug targets

In the previous two steps described, we identified the active compounds of the drugs. Then, the corresponding target proteins were identified with the TCMSP and ChemMapper databases, and the repeated targets were simultaneously eliminated. Finally, we used the UniProt KB database^5^ to standardize the target proteins and identify the confirmed human target genes.

### Identification of common targets for four Chinese patent medicine

Jvenn diagrams^6^ are widely used in biology to illustrate the differences between gene lists originating from different differential analyses (31). The targets of the four prescriptions were intersected, and the selected targets were identified as their common targets by default.

### Predicting putative targets of 2019-nCoV

The 2019-nCoV-related target proteins were screened from two databases: (1) the GeneCards (Ver. 4.14)^7^ database, the largest public platform, is used to effectively navigate gene–disease linkages (32, 33) and (2) the National Center for Biotechnology Information (NCBI)^8^ database is a popular choice for data deposition (34). We searched the two databases with the keyword “novel coronavirus” and set the species to “Homo sapiens.”

### Protein–protein interaction network construction and hub gene analysis

First, we intersected the shared targets of the four prescriptions with the genes associated with 2019-nCoV and obtained a Venn diagram of the intersecting gene symbols. Then, the potential targets were imported into STRING,^9^ which is a database for predicting protein–protein interactions. The STRING database defines PPIs and provides confidence ranges for data scores (high > 0.7; medium > 0.4; low > 0.15) (35). In this study, we limited the species to “Homo sapiens” and selected a confidence score > 0.7. Finally, we exported the downloaded “string_interactions.tsv” file and imported it into the Cytoscape 3.7.2^10^ platform to obtain and visualize the PPI network.

A hub gene is a gene that plays a crucial role in biological processes. In related pathways, the regulation of other genes is often affected by this gene. Therefore, the “cytoHubba”^11^ plug-in of Cytoscape was used to filter the hub nodes in the network according to the network topology parameters (36). The hub gene was further validated by cluster analysis using the MCODE plugin in Cytoscape.

### Gene ontology and pathway enrichment

Gene Ontology (GO) is an international standard system for classifying gene function (37). GO was used to describe the gene functions and the relationships between these concepts. GO divided gene functions into three aspects: molecular function (MF), cellular component (CC), and biological process (BP) (38).

Kyoto Encyclopedia of Genes and Genomes (KEGG) is a set of artificially drawn pathway maps representing molecular interactions and reaction networks and includes seven major biological processes, including metabolism, cellular processes, organismal systems, and so on.

To further investigate the multiple mechanisms of the potential targets in 2019-nCoV, we conducted of GO and KEGG Enrichment analyses using the clusterProfiler R package (ver. 3.6.3), which is an R package that automates the process of biological term classification and the enrichment analysis of gene clusters. Relevant pathways with *P*-values < 0.05 were selected (39). In addition, the Hub gene enrichment pathway with GO-BP was enriched *via* ClueGO.

### Network construction and analysis

To intuitively understand the common mechanism of the four recommended CPMs in the treatment of COVID-19 during the observation period, the identified active components, the potential targets, and the signal pathways with high scores were input into Cytoscape 3.7.2 software to construct the active ingredient—target—pathway network. At the same time, we constructed the relationship between the common active ingredients of four CPMs.

### Molecular docking

Angiotensin-converting enzyme 2 (ACE2) is the entry receptor for 2019-nCoV entry into host cells (40); S protein, a type I glycoprotein on the surface of the virus, plays a crucial role during virus entry into the host cells (41); 3-C like protease (3CL pro)play an important role in post-translational processing of replicase polyproteins (42). The crystal structure of the ACE2, S protein, 3CLpro was taken from the RSCB Protein Data Bank (PDB ID: 1R42, 6VSB, 6LU7). PyMol (version 2.3.4) software package was used to erase all the hetero atoms, water molecules, and inhibitor present in the structure (43). the non-covalent interaction of phytochemicals-protease was calculated using autodock Vina (version 1.1.2) software package for the docking analysis (44). Using this method, binding affinities of ligand-protease were determined and reported in kcal/mol unit. For the ACE2, grid box (82 Å × 68 Å × 78 Å) centered at (53.788, 60.568, 30.881) Å, for the 3CLpro, grid box (52 Å × 64 Å × 66 Å) centered at (−26.283, 12.599, 58.965) Å, for the S protein, and grid box (126Å × 126 Å × 126 Å) centered at (225.616, 226.49, 225.337) Å.

### Molecular dynamics simulation

To further investigate the stability of ligand small molecule binding proteins, molecular dynamics simulations (MDS) were performed using Gromacs 2020.6 software. Because the small ligand molecule is a multi-carbon ring skeleton structure, the Charmm36 force field with the TIP3P water model was chosen for simulation (see Supplementary Data Sheet 1 for specific parameters). The system temperature was set to 37°C for sodium chloride solution to simulate the real environment in the human body. During the MD simulation, all hydrogen bonds involved were constrained using the LINCS algorithm with an integration step of 2 fs. The electrostatic interactions were calculated using the Particle-mesh Ewald (PME) method with the cut-off value set to 1.2 nm. The non-key interaction truncation value is set to 10 Å and is updated every 10 steps. The protein-ligand complexes (hereafter referred to as complexes) are first pre-equilibrated for 100 ps to optimize the initial conformation of the protein in the solvent. After that, the NVT equilibrium of 100 ps with a coupling time constant of 0.1 ps was performed using a modified Berendsen temperature coupling algorithm to warm up the complex to 310 K with the solvent system. Subsequently, a 100 ps NPT equilibration was performed using a Berendsen constant pressure to equilibrate the solvent to the complex system to 1 bar. Ultimately, MDS of 50 ns duration was conducted to further investigate ligand binding relative to the receptor. Root Mean Square Deviation (RMSD) analysis was performed on the simulation results to analyze the stability of the ligand relative to the receptor during the simulation. The radius of gyration (Rg) can characterize the tightness of the protein structure and likewise relies on the radius of gyration to indicate the changes in the peptide chain relaxation of the protein during the simulation. In addition, hydrogen bond number analysis was performed for ligand and receptor binding to observe whether the binding is stable from the bonding perspective.

## Results

### The compounds, active ingredients and related targets of the four prescriptions

Based on the TCMSP database and the existing literature reports, 1,848, 1,818, 1,773, and 1,151 compounds of the four prescriptions HXZQ, JHQG, LHQW, and SFJD were retrieved, respectively. According to the criteria of OB ≥ 30% and DL ≥ 0.18, 203, 228, 241, and 179 active compounds and 269, 286, 246, and 259 predicted targets were identified, respectively. The number of the components, active components and potential targets of each prescription is shown in Table 2.

**TABLE 2:** Specific information of each prescription.

Prescription name	Number of compounds	Number of active ingredients	Number of predicted targets
HXZQ	1,848	203	269
JHQG	1,818	228	286
LHQW	1,773	241	246
SFJD	1,151	179	259

### Common targets for four prescriptions

**FIGURE 2:**
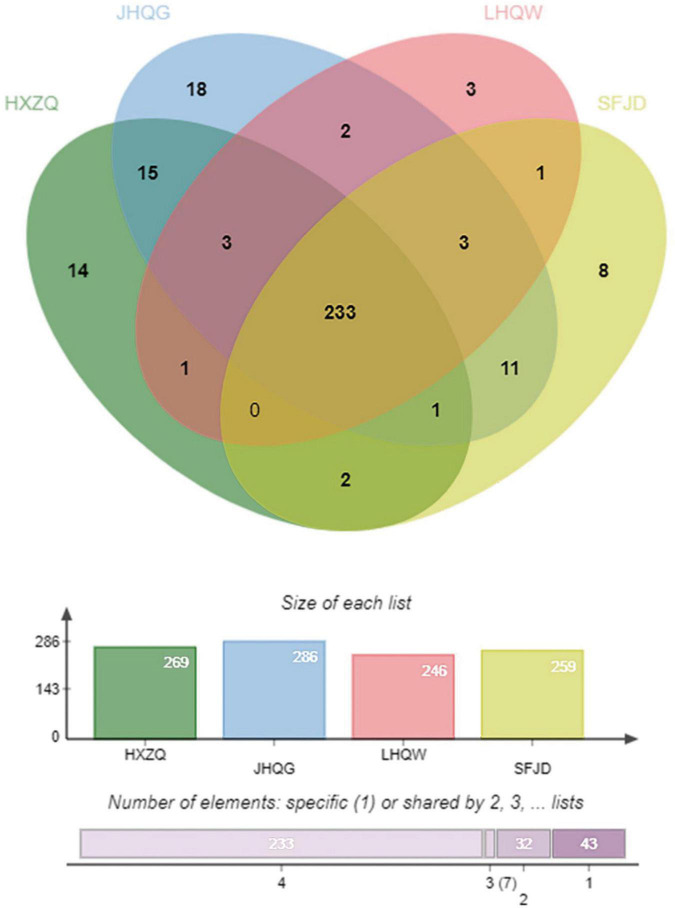
Common targets of the four prescriptions.

A total of 233 common targets of the four prescriptions were identified through jvenn diagrams, as shown in Figure 2.

### Protein–protein interaction network and hub gene analysis

2019-nCoV gene dataset collection from the NCBI and GeneCards databases, 48 potential targets and 350 potential targets in 2019-nCoV were identified, respectively, and 46 duplicates were eliminated. A total of 352 targets related to 2019-nCoV were identified.

**FIGURE 3:**
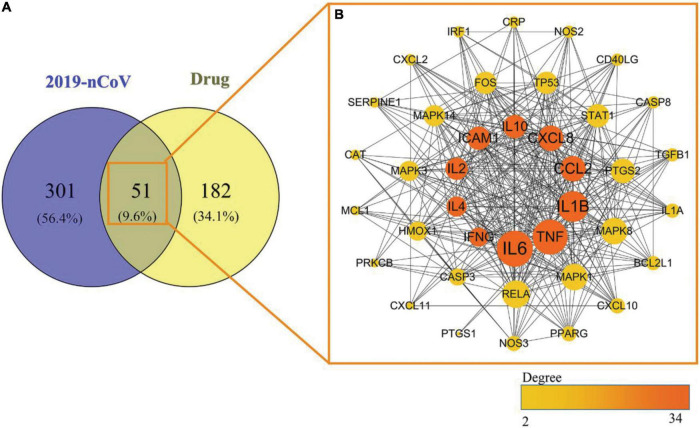
Mapping relationships between the four recommended CPMs and COVID-19. **(A)** Intersection targets of COVID-19 and four CPMs. **(B)** Protein interactions among 51 potential targets. The node’s size indicates the target’s degree, and the larger the node is, the higher the importance is. The thicker edges suggest stronger interactions. The degree value of nodes is positively correlated with node size and color brightness. The middle 10 targets are Hub genes screened based on the MCC method.

**FIGURE 4:**
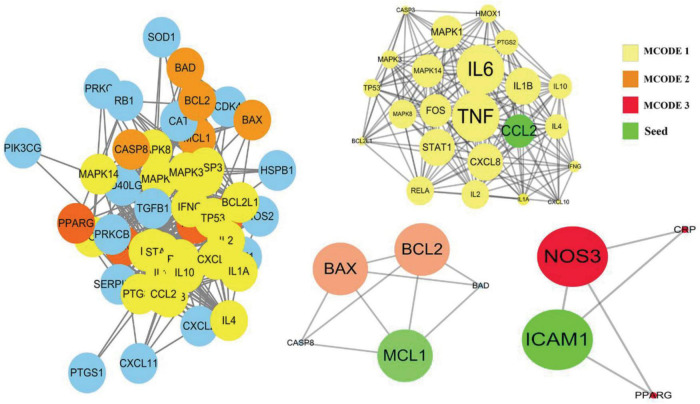
The hub genes were screened based on MCODE.

Among the above 352 2019-nCoV-related targets, the four prescriptions shared 51 common targets with the targets in 2019-nCoV (Figure 3A). The 51 putative therapeutic targets were imported into the STRING database to establish the putative therapeutic target PPI network, and then the “string_interaction.tsv” file was imported into Cytoscape 3.7.2 to perform network analysis. The screening of hub genes was performed using Cytoscape’s plugin cytoHubba and MCODE, respectively. The results of both screens were combined, and a total of 11 hub genes were obtained, namely TNF, IL6, IL1B, CXCL8, CCL2, IL2, IL4, ICAM1, IFNG, IL10, and MCL1. The hub genes screened by the plug-in cytoHubba and the plug-in MCODE are shown in Figures 3B, 4, respectively. The topological parameters of the 11 hub genes are shown in Table 3.

**TABLE 3:** The 11 hub nodes and their topological properties.

Rank	Name	Degree	Betweenness centrality	Closeness centrality	MCC score
1	TNF	33	0.046	0.77	2.02E + 07
2	IL6	34	0.061	0.783	2.02E + 07
3	IL1B	29	0.051	0.712	1.76E + 07
4	CXCL8	24	0.026	0.662	1.64E + 07
5	CCL2	24	0.023	0.671	1.56E + 07
6	IL2	21	0.013	0.644	1.51E + 07
7	IL4	19	0.005	0.603	1.39E + 07
8	ICAM1	22	0.021	0.653	1.19E + 07
9	IFNG	18	0.011	0.573	1.14E + 07
10	IL10	22	0.012	0.635	1.08E + 07
11	MCL1	11	0.002	0.547	

### Pathway and gene ontology term enrichment analysis

#### Gene ontology enrichment

**FIGURE 5:**
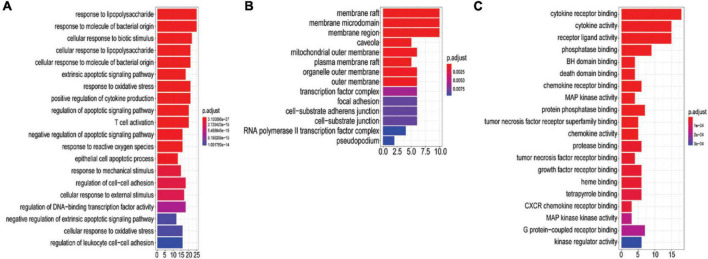
GO analysis of 51 potential targets genes. The *y*-axis shows the name of the GO entry, and the *x*-axis shows the value of gene number count, the value of which represents the number of targets enriched on the GO entry. The redder the color is, the smaller the value of p. adjust is. **(A)** The top 20 biological processes (BP). **(B)** GO-MF. **(C)** GO-CC.

A total of 396 GO-BPs, 55 GO-MFs, and 33 GO-CCs were enriched by R package (version 3.6.3). The top 20 GO terms according to *P*-values < 0.05 are shown in Figure 5. BP was mainly enriched in the regulation of the apoptosis process (5 in total), response to oxidative stress (3 in total), response to lipopolysaccharide (2 in total), response to molecules of bacterial origin (2 in total), inflammatory response, T cell activation and so on (see Figure 5A for details); CC was mainly concentrated in membrane rafts, membrane microdomains, membrane regions, mitochondrial outer membranes, organelle outer membranes, outer membranes, plasma membrane rafts, RNA polymerase II transcription factor complexes, cell-substrate adherens junctions, cell-substrate junctions and so on, and the membrane had the largest proportion (see Figure 5C); MF was mostly involved in inflammatory mediator cytokines, MAP kinase activity, phosphatase binding, heme binding, and so on (see Figure 5B).

#### Kyoto encyclopedia of genes and genomes enrichment

**FIGURE 6:**
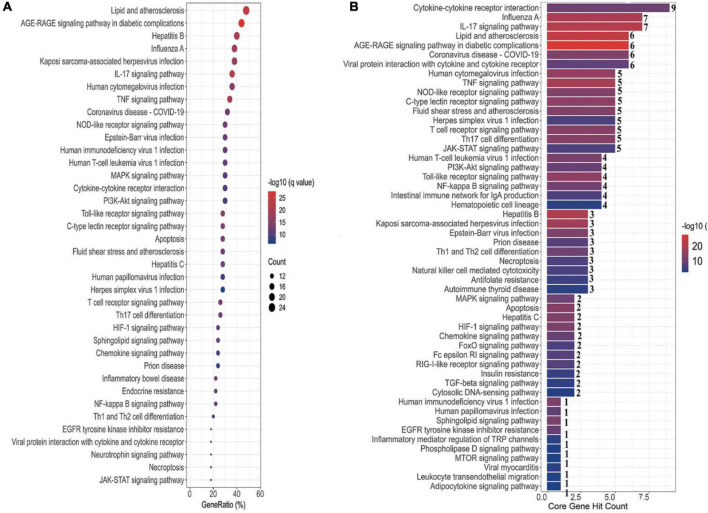
Pathway enrichment analysis and pathway hit to hub genes. **(A)** The top 39 signaling pathways associated with 51 potentially acting target genes. Pathways with a *P*-values < 0.05 were identified. The spot size represents the number of genes, and the color represents the *P*-value. The horizontal coordinate represents the generation, which is the ratio of the number of intersecting genes enriched in the pathway to the total intersecting genes; the vertical coordinate represents the name of the enriched pathway. **(B)** Signaling pathways associated with hub genes. The horizontal coordinate represents the number of hub gene hits on the pathway, and the vertical coordinate represents the pathway name.

To elucidate the signaling pathways involved in the treatment of COVID-19 with four CPMs, we subjected 51 drug and disease intersection targets to pathway enrichment analysis by R 4.1.3. A total of 166 pathways were enriched (*P* < 0.05), 92 of which may be associated with COVID-19. The first 39 signaling pathways were visualized and analyzed in Figure 6A. Figure 6B shows the enriched signaling pathways hitting hub genes, showing 53 pathways associated with the 11 hub genes. It further indicates that hub genes and enrichment pathways have some credibility. The specific parameters of the hub genes and their associated pathways of the four CPMs are shown in Table 4.

**TABLE 4:** Four CPMs hub genes and their associated pathways.

KEGG term	Count	%	Associated genes	*p*-adjust
Cytokine-cytokine receptor interaction	9	81.82	TNF, IL6, IL1B, CXCL8, CCL2, IL2, IL4, IFNG, IL10	5.35E-10
IL-17 signaling pathway	7	63.64	TNF, IL6, IL1B, CXCL8, CCL2, IL4, IFNG	2.23E-21
Influenza A	7	63.64	TNF, IL6, IL1B, CXCL8, CCL2, ICAM1, IFNG	1.92E-18
AGE-RAGE signaling pathway in diabetic complications	6	54.55	TNF, IL6, IL1B, CXCL8, CCL2, ICAM1	1.10E-27
Coronavirus disease—COVID-19	6	54.55	TNF, IL6, IL1B, CXCL8, CCL2, IL2	1.83E-12
Lipid and atherosclerosis	6	54.55	TNF, IL6, IL1B, CXCL8, CCL2, ICAM1	2.47E-23
Viral protein interaction with cytokine and cytokine receptor	6	54.55	TNF, IL6, CXCL8, CCL2, IL2, IL10	2.58E-08
JAK-STAT signaling pathway	6	54.55	IL6, IL2, IL4, IFNG, IL10, MCL1	1.31E-06
C-type lectin receptor signaling pathway	5	45.45	TNF, IL6, IL1B, IL2, IL10	9.90E-15
Fluid shear stress and atherosclerosis	5	45.45	TNF, IL1B, CCL2, ICAM1, IFNG	4.87E-13
Herpes simplex virus 1 infection	5	45.45	TNF, IL6, IL1B, CCL2, IFNG	2.48E-06
Human cytomegalovirus infection	5	45.45	TNF, IL6, IL1B, CXCL8, CCL2	5.48E-15
NOD-like receptor signaling pathway	5	45.45	TNF, IL6, IL1B, CXCL8, CCL2	1.09E-12
T cell receptor signaling pathway	5	45.45	TNF, IL2, IL4, IFNG, IL10	2.97E-13
Th17 cell differentiation	5	45.45	IL6, IL1B, IL2, IL4, IFNG	4.44E-13
TNF signaling pathway	5	45.45	TNF, IL6, IL1B, CCL2, ICAM1	1.13E-18
Hematopoietic cell lineage	4	36.36	TNF, IL6, IL1B, IL4	0.000561
Human T-cell leukemia virus 1 infection	4	36.36	TNF, IL6, IL2, ICAM1	1.23E-11
Intestinal immune network for IgA production	4	36.36	IL6, IL2, IL4, IL10	1.23E-06
NF-kappa B signaling pathway	4	36.36	TNF, IL1B, CXCL8, ICAM1	1.10E-10
PI3K-Akt signaling pathway	4	36.36	IL6, IL2, IL4, MCL1	5.77E-09
Toll-like receptor signaling pathway	4	36.36	TNF, IL6, IL1B, CXCL8	9.90E-15
Antifolate resistance	3	27.27	TNF, IL6, IL1B	6.27E-05
Autoimmune thyroid disease	3	27.27	IL2, IL4, IL10	0.00052
Epstein-Barr virus infection	3	27.27	TNF, IL6, ICAM1	3.49E-12
Hepatitis B	3	27.27	TNF, IL6, CXCL8	3.28E-20
Kaposi sarcoma-associated herpesvirus infection	3	27.27	IL6, CXCL8, ICAM1	1.66E-17
Natural killer cell mediated cytotoxicity	3	27.27	TNF, ICAM1, IFNG	2.76E-06
Necroptosis	3	27.27	TNF, IL1B, IFNG	1.15E-06
Prion disease	3	27.27	TNF, IL6, IL1B	1.92E-07
Th1 and Th2 cell differentiation	3	27.27	IL2, IL4, IFNG	6.18E-10
Apoptosis	2	18.18	TNF, MCL1	3.94E-13
Chemokine signaling pathway	2	18.18	CXCL8, CCL2	4.46E-09
Cytosolic DNA-sensing pathway	2	18.18	IL6, IL1B	0.000957
Fc epsilon RI signaling pathway	2	18.18	TNF, IL4	4.67E-07
FoxO signaling pathway	2	18.18	IL6, IL10	2.76E-06
Hepatitis C	2	18.18	TNF, IFNG	2.04E-12
HIF-1 signaling pathway	2	18.18	IL6, IFNG	8.80E-12
Insulin resistance	2	18.18	TNF, IL6	9.28E-05
MAPK signaling pathway	2	18.18	TNF, IL1B	5.20E-10
RIG-I-like receptor signaling pathway	2	18.18	TNF, CXCL8	5.56E-07
TGF-beta signaling pathway	2	18.18	TNF, IFNG	0.000463
Adipocytokine signaling pathway	1	9.09	TNF	0.0121
EGFR tyrosine kinase inhibitor resistance	1	9.09	IL6	3.41E-09
Human immunodeficiency virus 1 infection	1	9.09	TNF	6.67E-12
Human papillomavirus infection	1	9.09	TNF	2.27E-08
Inflammatory mediator regulation of TRP channels	1	9.09	IL1B	0.00054
Leukocyte transendothelial migration	1	9.09	ICAM1	0.007382
mTOR signaling pathway	1	9.09	TNF	0.00387
Phospholipase D signaling pathway	1	9.09	CXCL8	0.003141
Sphingolipid signaling pathway	1	9.09	TNF	2.41E-11
Viral myocarditis	1	9.09	ICAM1	0.0008

**FIGURE 7:**
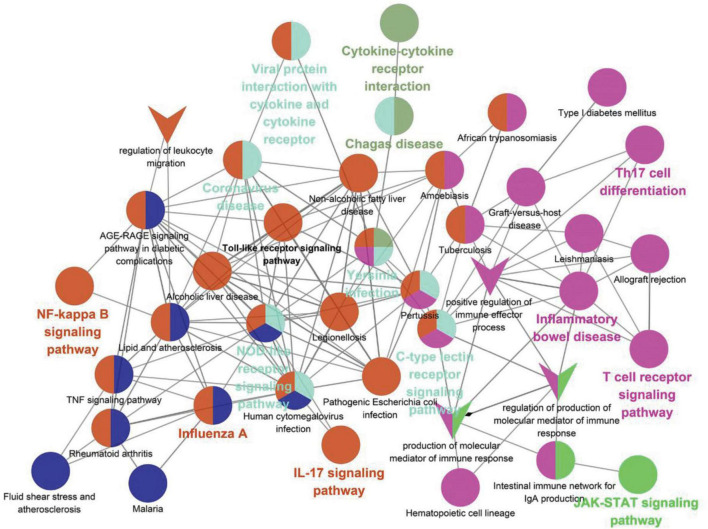
Correlation of KEGG enrichment of hub gene with GO-BP. The circle represents the pathway, and the “V” shape represents GO-BP. The node color means that the pathway is clustered into the same clusters with similar functions. The bolded pathway name means the *p*-value is smaller in that clusters with high confidence.

As shown in Table 5, the pathways were mainly enriched in three areas: (i) viral infections, such as coronavirus disease-COVID-19, and human cytomegalovirus infection. (ii) immunity, such as Toll-like receptor signaling pathway, T cell receptor signaling pathway, NOD-like receptor signaling pathway, and other signaling pathways; (iii) inflammation, such as cytokine-cytokine receptor interaction, IL-17 signaling pathway, Th17 cell differentiation, C-type lectin receptor signaling pathway, etc. In addition, the relationship between the Hub gene enrichment pathway and the gene ontology biological process (G0-BP) is shown in Figure 7. As shown in Figure 7, the pathways enriched by the hub genes, such as coronavirus disease, cytokine-cytokine receptor interaction, and viral protein interaction with cytokine and cytokine receptor, are closely related to the production and regulation of molecular mediators of the immune response, the positive regulation of immune effector processes, and the regulation of leukocyte migration.

**TABLE 5:** Classification of the top 39 related pathways.

Classification	Signaling pathways
Anti-viral effect	Hepatitis B, Influenza A, Kaposi sarcoma-associated herpesvirus infection, Human cytomegalovirus infection, Coronavirus disease—COVID-19, Hepatitis C, Epstein-Barr virus infection, Human immunodeficiency virus 1 infection, Human T-cell leukemia virus 1 infection, Human papillomavirus infection, Viral protein interaction with cytokine and cytokine receptor, Prion disease
Inflammatory response	IL-17 signaling pathway, TNF signaling pathway, C-type lectin receptor signaling pathway, Th17 cell differentiation
Regulation of immune system	Toll-like receptor signaling pathway, T cell receptor signaling pathway, NOD-like receptor signaling pathway, Th1 and Th2 cell differentiation, Chemokine signaling pathway, Fc epsilon RI signaling pathway

### Network construction and analysis

#### Active component-common active component network of four prescriptions

**FIGURE 8:**
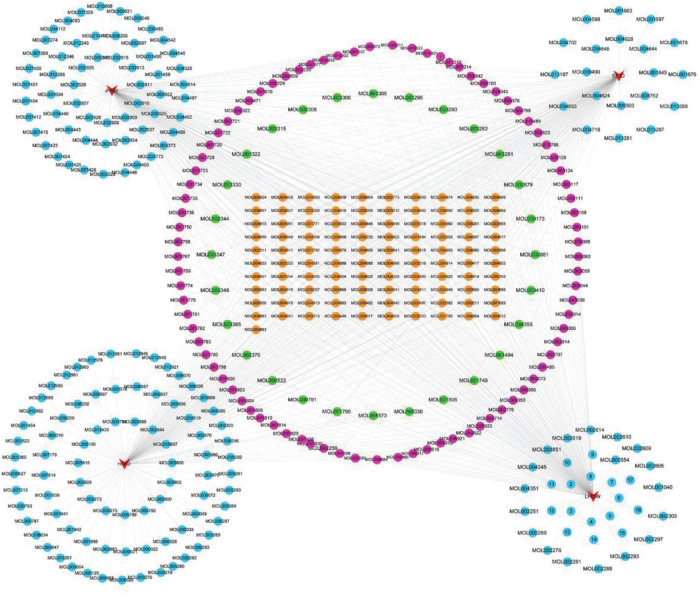
Active component-common active component network of the four prescriptions. The red triangles represent the four prescriptions; the Orange, green, rose red, and sky blue circles represent the common active ingredients of the four prescriptions, the common active ingredients of three prescriptions, two prescription common active ingredients, and each prescription unique active ingredient.

There are 411 nodes and 850 edges in the active component-common active component network of the four prescriptions (Figure 8). The details of the 101 common active components are provided in Supplementary Table 1.

#### Active component-potential target–pathway network

**FIGURE 9:**
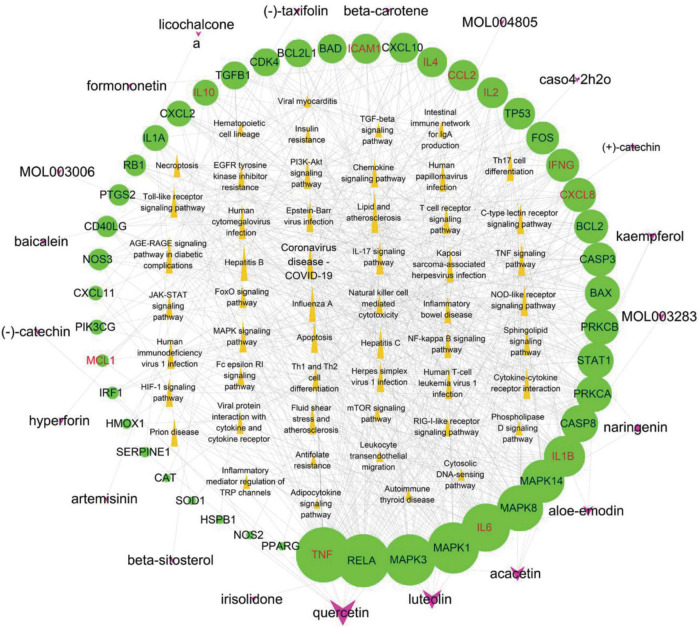
Potential active component-potential target–pathway network. Green circles represent action targets, yellow triangles represent the 53 signaling pathways associated with Hub genes, and rosy V shapes represent the four prescribed active ingredients. The node size is positively related to the degree, and the edges represent the interactions between the nodes.

The 53 signaling pathways associated with hub genes, potential targets, and active ingredients in the signaling pathways were entered into Cytoscape 3.7.2 software to construct the active ingredient-potential target-pathway network, as shown in Figure 9. According to the network diagram and topological parameters, it can be seen that the 21 active ingredients, 46 targets, and 56 signaling pathways are closely related to each other. The greater the nodal degree, the more biological functions it involves, indicating its biological importance. On the one hand, considering that fewer key components were screened by degree greater than the mean value, and on the other hand, we screened the pathway three times to further ensure the integrity and reliability of the study, all the active ingredients enriched this time were initially used as key active ingredients. Subsequently, key components of the initial screen were excluded if they had docking binding energies greater than −5 kcal/mol to the three potential action targets of COVID-19. Twenty-one key compounds were finally identified, and their specific parameters and sources are shown in Table 6.

**TABLE 6:** Pharmacokinetics and sources of the top 21 key active ingredients.

Mol ID	Molecule name	Degree	OB. (%)	DL	Source (abbreviations of latin names)
MOL000098	Quercetin	10	46.43	0.28	PC, GU, ZJ, LJ, ES, FS, AC, HC, RJ, Patriniaceae, VO, BC, RR, FT
MOL000006	Luteolin	7	36.16	0.25	PF, PG, LJ, ES, FS, AC, MH, RJ, Patriniaceae, VO, RR, GU
MOL001689	Acacetin	5	34.97	0.24	PG, SB, MH, IT, Patriniaceae
MOL000471	Aloe-emodin	3	83.38	0.24	MH, RP
MOL000422	Kaempferol	2	41.88	0.24	GU, FS, AA, AI, AC, LJ, FS, ES, CF, HC, Patriniaceae, VO, BC, RR, FT
MOL003283	(2R, 3R, 4S)-4-(4-hydroxy-3-methoxy-phenyl)-7-methoxy-2,3-dimethylol-tetralin-6-ol	2	66.51	0.39	FS
MOL004328	Naringenin	2	59.29	0.21	GU, CR, MH, GU, ES
MOL005916	Irisolidone	1	37.78	0.3	PC
MOL000358	Beta-sitosterol	1	36.91	0.75	PT, PF, ZJ, ZO, SB, FS, FT, AI, LJ, ES, RP, RJ, Patriniaceae, VO
MOL007424	Artemisinin	1	49.88	0.31	AC
MOL003347	Hyperforin	1	44.03	0.6	FS
MOL000096	(−)-catechin	1	49.68	0.24	ZJ, Rheum
MOL002714	Baicalein	1	33.52	0.21	PT, SB, GU
MOL003006	(−)-(3R,8S,9R,9aS,10aS)-9-ethenyl-8-(beta-D-glucopyranosyloxy)-2,3,9,9a,10,10a-hexahydro-5-oxo-5H,8H-pyrano[4,3-d]oxazolo[3,2-a]pyridine-3-carboxylic acid_qt	1	87.47	0.23	LJ
MOL000392	Formononetin	1	69.67	0.21	GU
MOL000497	Licochalcone a	1	40.79	0.29	GU, PC
MOL001736	(−)-taxifolin	1	60.51	0.27	Isatis
MOL002773	Beta-carotene	1	37.18	0.58	PF, ZJ, LJ, AL, LJ, VO
MOL004805	Shinflavanone	1	31.79	0.72	GU
caso4⋅2h2o	Caso4⋅2h_2_o	1	/	/	GF
MOL000492	(+)-catechin	1	54.83	0.24	PF, ZJ, ES, PA, ES, PA, RJ

The Latin names of the four PCM constituent drugs can be referred to as shown in Table 1.

### Molecular docking

Lopinavir, remdesivir, ritonavir, ribavirin have been widely used in antiviral therapy (45–47), mainly through the control of their binding sites. In Autodock Vina scoring system, lower binding score indicates the better docking interaction between ligand and protein. According to the analysis of docking results (Table 7), binding affinities of the phytochemicals are distributed within the range of −7.0 to −9.1 kcal/mol, Similar to anti-viral drugs within the range of −6.1 to −10.4 kcal/mol. Molecular docking is performed to find out the best candidates among the 9 phytochemicals based on their binding scores. In docking with 3CLPro, ACE2, and S protein, the affinity of shinflavanone was in the order of −8.4, −9.7, and −10.4 kcal/mol, which were superior to these four antiviral drugs. In addition, the beta-carotene binding energy in docking with ACE2 was lower than that of Ritonavir and Ribavirin. In docking with S protein, beta-carotene and beta-sitosterol binding energy were lower than Remdesivir, Ribavirin, and Ritonavir; hyperforin and baicalein binding energy was lower than Ribavirin and Ritonavir.

**TABLE 7:** Chemical information and binding energy of four CPM main potential active ingredients and antiviral drugs.

Compounds	Structure	CAS ID	MF	MW	Affinity/ (kcal/mol)	Affinity/ (kj/mol)
Quercetin	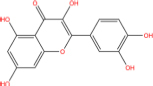	117-39-5	C15H10O7	302.24	−7.4	−30.932^➀^
					−8	−33.440^➁^
					−8.2	−34.276^➂^
Luteolin	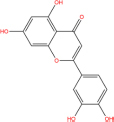	491-70-3	C15H10O6	286.24	−7.3	−30.514^➀^
					−8	−33.440^➁^
					−8.3	−34.694^➂^
Acacetin	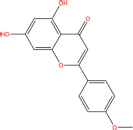	480-44-4	C16H12O5	284.26	−7.3	−30.514^➀^
					−7.6	−31.768^➁^
					−7.7	−32.186^➂^
Aloe-emodin	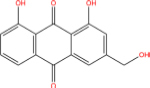	481-72-1	C15H10O5	270.24	−7.4	−30.92^➀^
					−7.8	−32.604^➁^
					−8	−33.44^➂^
Kaempferol	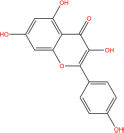	520-18-3	C15H10O6	286.25	−7.8	−32.604^➀^
					−7.6	−31.768^➁^
					−8.1	−33.858^➂^
(2R,3R,4S)-4-(4-hydroxy-3-methoxy-phenyl)-7-methoxy-2,3-dimethylol-tetralin-6-ol	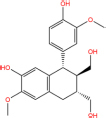	548-29-8	C20H24O6	360.4	−6.2	−25.916^➀^
					−7.3	−30.514^➁^
					−8	−33.44^➂^
Naringenin	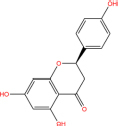	67604-48-2	C15H12O5	272.25	−7.1	−29.678^➀^
					−8.2	−34.276^➁^
					−8.3	−34.694^➂^
Irisolidone	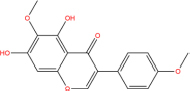	2345-17-7	C17H14O6	314.29	−7.4	−30.932^➀^
					−6.9	−28.842^➁^
					−7.4	−30.932^➂^
Beta-sitosterol	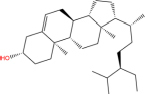	83-46-5	C29H50O	414.71	−7.5	−31.350^➀^
					−7.8	−32.604^➁^
					−9.1	−38.038^➂^
Artemisinin	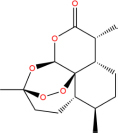	63968-64-9	C15H22O5	282.33	−7.2	−30.096^➀^
					−7.2	−30.096^➁^
					−8.4	−35.112^➂^
hyperforin	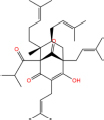	11079-53-1	C35H52O4	536.8	−6.1	−25.498^➀^
					−7.7	−32.186^➁^
					−8.6	−35.948^➂^
(−)-catechin	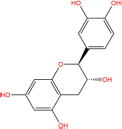	18829-70-4	C15H14O6	290.27	−7.3	−30.514^➀^
					−7.7	−32.186^➁^
					−8.3	−34.694^➂^
Baicalein	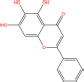	491-67-8	C15H10O5	270.23	−7.7	−32.186^➀^
					−7.8	−32.604^➁^
					−8.5	−35.530^➂^
MOL003006	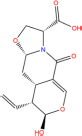			281.29	−6.5	−27.17^➀^
					−6.7	−28.006^➁^
					−7.5	−31.35^➂^
Formononetin	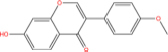	485-72-3	C16H12O4	268.26	−7.2	−30.096^➀^
					−7.4	−30.932^➁^
					−7.5	−31.35^➂^
Licochalcone a	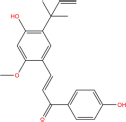	58749-22-7	C21H22O4	338.4	−7.4	−30.932^➀^
					−8	−33.440^➁^
					−7.7	−32.186^➂^
(−)-taxifolin	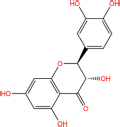	111003-33-9	C15H12O7	304.25	−7.4	−30.932^➀^
					−7.7	−32.186^➁^
					−7.9	−33.022^➂^
Beta-carotene	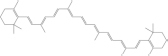	7235-40-7	C40H56	536.9	−7.5	−31.35^➀^
					−7.8	−32.604^➁^
					−9.6	−40.128^➂^
Shinflavanone	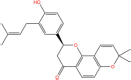	157414-03-4	C25H26O4	390.5	−8.4	−35.112^➀^
					−9.7	−40.546^➁^
					−10.4	−43.472^➂^
(+)-catechin	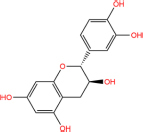	154-23-4	C15H14O6	290.27	−7.1	−29.678^➀^
					−7.8	−32.604^➁^
					−8.1	−33.858^➂^
Remdesivir	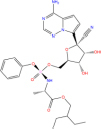	1809249-37-3	C27H35N6O8P	602.58	−8.2	−34.276^➀^
					−9	−37.620^➁^
Ribavirin	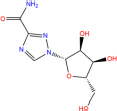	36791-04-5	C8H12N4O5	244.2	−7	−29.260^➀^
					−7.4	−30.932^➁^
					−8.4	−35.112^➂^
Ritonavir	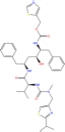	155213-67-5	C37H48N6O5S2	720.96	−7.9	−33.022^➀^
					−8.5	−35.530^➁^
					−7.8	−32.604^➂^
Lopinavir	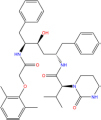	192725-17-0	C37H48N4O5	628.8	−8.3	−34.694^➀^
					−9	−37.620^➁^
					−10	−41.800^➂^

➀: Binding energy with 3CL hydrolase; ➁: Binding energy with ACE2; ➂: Binding energy to S-protein. MOL003006: (−)−(3R,8S,9R,9aS,10aS)-9-ethenyl-8-(beta-D-glucopyranosyloxy)-2,3,9,9a,10,10a-hexahydro-5-oxo-5H,8H-pyrano[4,3-d]oxazolo[3,2-a]pyridine-3-carboxylic acid_qt.

**FIGURE 10:**
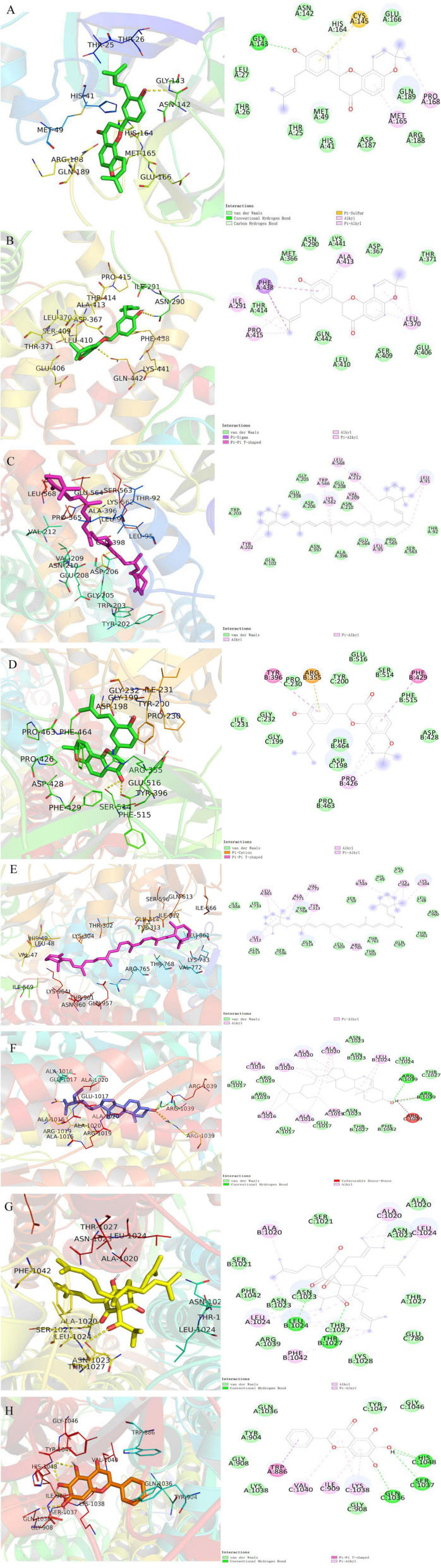
Molecular models of the selected compounds binding to the target proteins. **(A)** The docking mode and interactions between shinflavanone and 3CL. **(B–C)** Shinflavanone, beta-carotene and ACE2. **(D–H)** Shinflavanone, beta-carotene, beta_sitosterol, hyperforin, baicalein and S protein.

Additionally, the docking analysis between the selected compounds and the target proteins was shown in Figure 10. In docking with 3CLPro, the compound shinflavanone formed hydrogen bonds with GLY-143 to interact with each other (Figure 10A). In docking with S proteins, β-sitosterol formed three hydrogen bonds with ARG-1039 (A), ARG-1039 (B), and ARG-1039 (C), respectively (Figure 10F). Hyperforin forms two hydrogen bonds with LEU-1024 (B) and THR-1027 (B), respectively (Figure 10G). Baicalein formed two hydrogen bonds with GLN-1036(C) and HIS-1048(C), respectively (Figure 10H). These 21 phytochemicals bind to the active site of the major protease of 2019-nCOV with lower binding energy than the crystal structured ligand, implying that they may have strong antiviral activity.

### Molecular dynamics simulation

**FIGURE 11:**
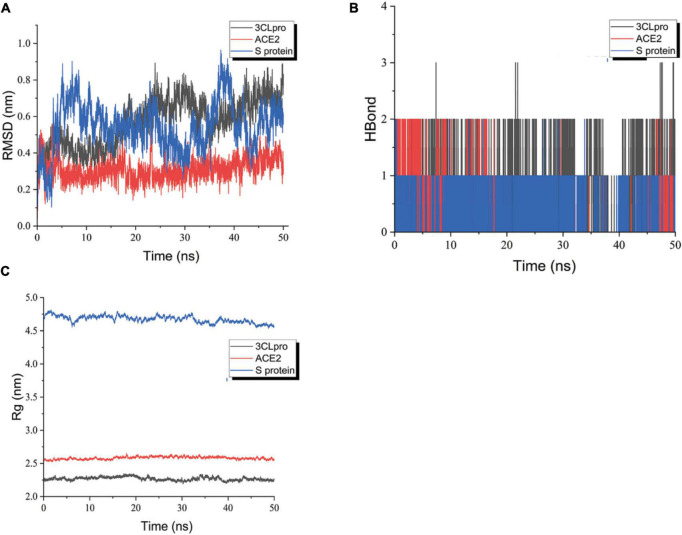
RMSD plot during molecular dynamics simulations. **(A)** The RMSD of ligand-receptor. **(B)** The H-Bond of ligand-receptor. **(C)** The Rg of ligand-receptor. Blue polygonal line means shinflavanone and S protein, red polygonal line means shinflavanone and ACE2, black polygonal line means shinflavanone and 3-C like protease.

From the RMSD plot analysis (Figure 11A), it is observed that the complex formed by the compound and ACE2 protein was more stable during the simulated 50 ns. The RMSD values of the ligand relative to the receptor were maintained in a low range and fluctuated less, suggesting that the ligand did not move much relative to the receptor and the binding was more stable. The RMSD of the compound relative to 3-C like protease maintained an increasing trend in the first 25 ns or so, and showed a trend of decreasing and then increasing to the RMSD value at 25 ns from 25 to 40 ns, and basically maintained equilibrium with less fluctuation in the last 10 ns. The possible reason was that the ligand shifted the binding cavity of the protein as the simulation proceeded. After entering the new binding cavity and undergoing particular rotation, it maintained a stable conformation and conformation in the last 10 ns, forming a more stable binding. The stability of the binding can be further verified by extending the duration of the kinetic simulation later. The RMSD values of the compounds relative to the S proteins kept fluctuating widely during the simulation, suggesting that the binding between the ligand and the receptor may not be very stable. Therefore, further extension of the kinetic time is needed to investigate the binding stability.

Rg can characterize the tightness of the protein structure, and the same radius of gyration can be relied on to distinguish the changes in the peptide chain relaxation of the protein during the simulation. As shown in Figure 11C, the Rg of both 3CLpro and ACE2 proteins remained relatively stable throughout the simulation. The Rg of 3CLpro protein was basically maintained around 2.25 nm, and that of ACE2 protein was basically maintained around 2.5 nm, suggesting that the complexes formed by the proteins and ligands were more stable. However, the Rg of S protein shows some fluctuations, which may be related to the fact that the small molecule is constantly undergoing conformational and conformational transitions, which in turn have some effects on the protein structure. It is in agreement with the previous analysis of RMSD and RMSF.

The number of hydrogen bonds between the protein and the ligand varies with time during the simulated 50 ns. As can be seen in Figure 11B, the 3-C like protease maintains the formation of more than two hydrogen bonds between the ligand and the receptor for nearly half of the simulation. Several hydrogen bonds between the ACE2 protein and the ligand were always formed. The overall situation was similar to that of 3-C like protease. The S protein basically maintains a hydrogen bond with the ligand during the simulation, suggesting that the ligand may always keep a part of the molecule relatively static in conformation and conformation during the simulation. In contrast, the rest of the molecule undergoes conformational and conformational transitions.

In general, the ligand maintains a more stable binding to the 3-C like protease and ACE2 protein throughout the simulation. In the complexes formed with S proteins, the ligands kept undergoing conformational and conformational transitions. The stability of S protein-ligand binding can be further explored later by extending the kinetic simulation time.

## Discussion

Traditional Chinese medicine (TCM) and herbal medicines (HMs) have a long history and play an indispensable role in the prevention and treatment of several epidemic diseases (48). Based on network pharmacology and systems biology, this paper discusses the possible common mechanisms of the four CPMs recommended by the *Diagnosis and Treatment Protocol for COVID-19* during the Medical Observation period.

### The material basis of the medicinal effect of traditional Chinese medicine

These four CPMs have been widely used and well-established in the Chinese protocol for treating COVID-19. At the same time, the specific content and proportion of the ancient books from which the CPM is derived are described for convenience of reference. The research on the material basis of the efficacy of TCM is gradually rising from molecular biology to the level of systems biology. Although there is no clear definition of the material basis of pharmacological efficacy in Chinese medicine, different theories and hypotheses exist, such as the all-component theory, the active ingredient theory, the metabolic component, and the multi-component synergism theory (49). However, most scholars believe that TCM’s effectiveness is reflected in the overall biological effect of the entire chemical composition, which is the synergistic effect of multiple components, not a single chemical entity. It is because a single ingredient does not represent an herb’s drug properties and efficacy, nor is the efficacy the sum of all ingredients (50). In drug compatibility, there will be physical or chemical reactions, which will lead to the formation of new compounds, affect the dissolution rate of effective and toxic components in the formula, or have no noticeable effect. In addition, the chemical components that act on multiple targets and produce overall effects after entering the human body may originate from inherent components of the herbs, products formed during the preparation process, or metabolites produced by the interaction between the drug and the body after entering the body (51). Lan Wang et al. demonstrated synergistic effects between three active compounds isolated from three components of a TCM formulation in an animal model of acute promyelocytic leukemia (APL) and an APL cell line (52). At present, the existing methods to study the synergistic effect of Chinese herbal medicines include mathematical modeling (such as CI) and computer technology (such as systems biology analysis, S2S), fractionation technology combined with CI models, and combination of effective components of traditional Chinese medicines (50). In addition, in 2008, China launched the 15-year “Herbalome Project,” which aims to elucidate the chemical composition, structure, and function of commonly used Chinese herbal medicines and Chinese medicine formulations, establish a standard resource library and explain the synergistic and complementary mechanisms of multi-component Chinese medicines at multiple targets (53, 54). Although the synergistic research of Chinese herbal medicine is still in its infancy, we believe that it will develop better and better with the efforts of everyone.

### Active compounds analysis

According to the “active ingredient-potential target-pathway,” 21 essential compounds were identified, followed by quercetin, luteolin, acacetin, aloe-emodin, kaempferol, naringenin, irisolidone, beta-sitosterol, artemisinin, hyperforin, etc. Most of them are flavonoids. Flavonoids are natural antiviral compounds with a wide range of antiviral activity, anti-inflammatory, antioxidant, and immunomodulatory effects (55–57). Molecular docking results showed that shinflavanone had a better affinity for SARS-CoV-2 3CL hydrolase, ACE2, and S protein than all four commonly used antiviral drugs, suggesting that it may be superior in interfering with virus entry into host cells and viral replication. In addition, 19 compounds also showed better affinity to SARS-CoV-2 3CL hydrolase or ACE2 or S protein than one of these four commonly used antiviral drugs, such as quercetin, luteolin, acacetin, aloe-emodin, kaempferol, naringenin, beta- sitostero, artemisinin, hyperforin, baicalein, formononetin, licochalcone A, (−)-taxifolin, beta-carotene, (+)-catechin, irisolidone, and (−)-catechin, suggesting that TCM has excellent potential in the treatment of SARS-CoV-2.

(I). Flavonoids

Quercetin has many effects, such as anti-inflammatory, immunomodulatory, antioxidant, and free radical scavenging, which can play a prominent therapeutic role in lung injury (58–63). Quercetin can block the enzymatic activity of coronavirus 3cl protease in treating viral infections such as Sars-CoV infection. Quercetin also blocks the entry of the viral receptor complex into the cell and interrupts its life cycle, leading to viral death (64). In addition, as an essential consideration in developing new antiviral drugs (65, 66), quercetin can increase the bioavailability of antiviral drugs in the body. Luteolin has potent anti-inflammatory, antiviral, antibacterial, and other effects (67, 68). At the lowest concentration (1 mol/L), it can effectively reduce the expression of NF-KB P65, IL-1β, IL 6, and TNF-α in anti-inflammatory (69). Kaempferol has antiviral, anti-inflammatory, antioxidant, and other effects (70, 71). In addition, some studies reported that quercetin, luteolin, and kaempferol could inhibit the proteolytic activity of Sars-CoV 3CLpro (52–54). Naringenin is famous for its antiviral effect and has anti-inflammatory, antioxidant, and immunomodulatory effects (72–75). In addition, naringenin can inhibit inflammatory factors through the NF-κB pathway and reduce LPS-induced lung inflammation. Licochalcone A has anti-inflammatory, immune-promoting, and other biological effects (76, 77). It has been reported in the Lancet that the main active ingredient of licorice (glycyrrhizic acid) effectively inhibits the replication of clinical isolates of sars viruses, and licochalcone A may have the same function (78). Shinflavanone is also derived from Glycyrrhiza glabra, and its antiviral may be superior according to molecular docking, but it is relatively little studied at present and needs further research.

Baicalein and acacetin are flavonoids in scutellaria baicalensis, with anti-inflammatory, antioxidant, and ameliorative effects on acute lung injury (79). Baicalein strongly inhibits virus replication and improves the acute lung injury induced by influenza A and B viruses (80). The synergistic activity of baicalein and ribavirin against influenza virus infections in cell culture and mice was investigated by Chen, and it was discovered that, compared with ribavirin alone, the combination of baicalein and ribavirin increased the survival rate in mice infected with a lethal dose of H1N1 influenza virus (81). Furthermore, baicalein and acacetin can significantly reduce airway hyperresponsiveness, inflammation, and mucus production (82, 83). Acacia can resist plasmodium and regulate immunity (84). Most key compounds have antiviral, anti-inflammatory, and immune-regulating effects. Therefore, it can be inferred that they treat 2019-nCoV mainly through anti-inflammatory, antiviral, and immune-regulating activities.

(II). Others

Flavonoids and terpenoids are natural antiviral compounds. Artemisinin is a commonly used anti-malarial terpenoid compound with a broad-spectrum antiviral activity that has recently been repurposed as a potential COVID-19 drug. Recent studies have confirmed the antiviral activity of artemisinin and its derivatives against SARS-2-CoV-2 at micromolar concentrations (85, 86). Several clinical trials have shown that artemisinin-related agents can help improve SARS-CoV-2 clearance, shorten the time to conversion of SARS-CoV-2 tests to negative, improve the patient condition, and contribute to faster recovery of COVID-19 patients (87, 88). In addition, artemisinin has anti-inflammatory activity and reduces systemic levels of inflammatory cytokines that contribute to cytokine storm and inflammatory organ damage in high-risk COVID-19 patients (89). Aloe-emodin can impede coronaviruses’ entry, replication, and release (90–92). In molecular docking, aloe-emodin has an excellent binding ability to the main protease (6LU7) and nucleocapsid phosphoprotein of COVID-19. It can be used as a potential inhibitor of anti-COVID-19 main protease and a potential drug to cure SARS-CoV-2 infection (93, 94).

### Target analysis of traditional inhibitors and four Chinese patent medicines

According to the current status of COVID-19 therapy, the targets of COVID-19 inhibitors involve four main areas, which are briefly described as follows.

(1) Antiviral drugs: SARS-CoV-2 main protease inhibitors (95–98), HIV protease inhibitors (99), RNA-dependent RNA polymerase inhibitors (100).
(2) Cytokine monoclonal antibodies/inhibitors: IL6 monoclonal antibodies, complement component 5 inhibitors, PD-1 blocking antibody, human granulocyte-macrophage colony-stimulating factor receptor inhibitors, IL17A antagonist, IL1β antibody, vascular endothelial-derived growth factor antibody, IL1 receptor antagonist, anti-C5a receptor antibody, and tumor necrosis factor-α inhibitor (101).
(3) Interferons: IFN-α1b Eye Drops, IFN-β1b, IFN-β1a, IFN atomization, IFN-α1b spray, recombinant super-compound IFN, and IFN aerosol inhalation (101).
(4) Others: RAAS inhibitors (102, 103), JAK inhibitors (104), cathepsin L-selective inhibitors, and proton pump inhibitors (105).

At the same time, TCM differs from chemical drugs in that they usually have simple and straightforward targets of action, while CPMs consist of multiple herbs with multiple targets, and their specific targets of action are relatively complex (106). However, the therapeutic targets of the four CPM in the treatment of COVID-19 protocal are consistent with traditional COVID-19 inhibitors, which further confirms its feasibility. The targets of action of four CPMs for the treatment of COVID-19 are reviewed below. Some of them may still be in the theoretical stage, pending further basic experiments.

➀ LHQW

Nanshan Zhong et al. infected Vero-E6 cells and Huh-7 cells with clinically isolated SARS-CoV-2 virus and assessed the antiviral activity of LHQW against SARS-CoV-2 using CPE and plaque reduction assays, and measured the expression levels of pro-inflammatory cytokines in Huh-7 cells by real-time quantitative PCR. The results showed that LHQW significantly inhibited SARS-CoV-2 replication in Vero E6 cells and reduced the production of pro-inflammatory cytokines such as IL-6, TNF-α, CCL-2/MCP-1, and CXCL-10/IP-10 (13).

➁ JHQG

JHQG is the first TCM prescription for the treatment of H1N1 infection. Its potential mechanism of action for the treatment of COVID-19 is anti-inflammatory, anti-SARS-CoV-2 (107, 108), mainly through the regulation of interleukin receptors (IL-1A, IL-1B, IL-2, IL-4, IL-6, IL-10), mitogen-activated protein kinase receptors (MAPK1, MAPK3, MAPK8, MAPK14), TNF and other significant targets, thus achieving the treatment of COVID-19 (109–112). JHQG is derived from the combined formula of Yin Qiao San (YQS) and Ma Xing Shi Gan Tang (MXSG) with addition and reduction. Cellular experiments have shown that MXSG can regulate the expression and activation level of the Toll-like receptor (TLR) in the immune response pathway, which regulates the secretion and protein expression of IFN and effectively fights against viruses (113). Animal experiments showed that YQS could antagonize the function of influenza virus non-structural protein NS1, inhibit the expression of the JAK/STAT signaling pathway and promote the production of antiviral protein (Mx A) induced by IFN-α (114).

➂ HXZQ

The potential mechanism of action of HXZQ for COVID-19 is inhibiting SARS-CoV-2 replication, improving immune, and anti-inflammatory, mainly by acting on 3CLpro (115), PTGS2, AR, HSP90AB1, CAMSAP2, PPARG, NOS2, and other targets (116–118).

➃ SFJD

The potential mechanism of action of SFJD for the treatment of COVID-19 is anti-inflammatory, anti-SARS-CoV-2, mainly acting on mitogen-activated protein kinase receptors (MAPK1, MAPK3, MAPK8, MAPK14), interleukin receptors (IL-6, IL-10), TNF, PTGS2, CASP3, and other multiple targets to achieve the therapeutic effect on COVID-19 (119, 120). In SFJD alone or combined with oseltamivir for the treatment of chronic obstructive pulmonary disease induced by influenza A virus, SFJDC alone and combined with oseltamivir were found to control airway inflammation and lung injury in IAV-infected rats by modulating NLRP3 inflammatory vesicles and subsequently downregulating IL-1β and IL-18 (15). The results of experiments in yeast thermogenesis model rats confirmed the significant antipyretic effect of SFJD, which can significantly inhibit the production of inflammatory factors PGE2, thus considerably inhibiting the production of thermogenic cytokines TNF-α, IL-1α, IL-1β, IL-6. It reduces the amount of thermogenic factors cAMP, Na+, K+-ATPase, decreases cAMP/cGMP, reduces heat production, and increases the amount of endogenous antipyretic mediator AVP, thus exerting its antipyretic effect (121). The immunomodulatory effect of SFJD on rats with pneumonia was further verified in a rat model of pneumonia caused by streptococcus pneumonia. It has a therapeutic impact on pneumonia model rats by reducing B lymphocytes, CD8+ ratio, and elevated levels of IL-1α, IL-1β, IL-2, IL-10, TNF-α, IFN-α, IFN-γ, IgM, IgG, CD4+/CD8+, and NK cells (122).

### Analysis of the common action targets of four Chinese patent medicines

In the post-genetic era, researchers have recognized that many high-incidence diseases involve multifactorial physiological and pathological factors (123). Multi-target or multi-component therapeutics will receive increasing attention, and traditional Chinese medicine may become an essential resource for multi-component multi-target drug design (124). In this study, a total of 11 hub genes were identified through two plugins in cytoscape, cytoHubba, and Cluego. These hub genes are primarily inflammatory mediator cytokines, which are consistent with four CPM targets, including interleukins (IL-2, IL-4, IL-6, CXCL8, IL-10, and IL-1B), interferon (IFNG), tumor necrosis factor (TNF), chemokine (CCL2), and adhesion molecule (ICAM1). A study published in the Lancet notes that the occurrence and development of 2019-nCoV are closely related to cytokine storms. Cytokine storms are severe systemic reactions caused by the overactivation of the immune system by infection, drugs, or certain diseases, which can lead to multiple organ failure and even death (125). In patients with severe and critical COVID-19, the immune system is disrupted, thereby producing large amounts of inflammatory cytokines, which leads to life-threatening cytokine storms (126). Therefore, it is necessary to detect and interfere with inflammatory cytokines as soon as possible and initiate immunotherapy, aiming to avoid the progression of mild and common infections to severe and critical disease. This necessity further demonstrates the feasibility of the four prescriptions for the treatment of 2019-nCoV.

### Pathway analysis

**FIGURE 12:**
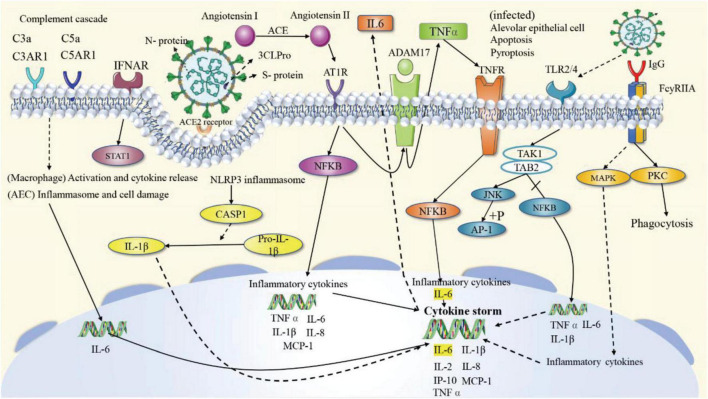
The specific mechanisms of action of the four CPMs on the Coronavirus disease—COVID-19 pathway. The solid arrows represent activation, the dashed arrows represent indirect effects, and the vertical lines above the arrows represent inhibition.

According to the KEGG enrichment analysis results of the potential targets, it can be inferred that the four prescriptions can achieve therapeutic effects against COVID-19 by targeting antiviral, inflammatory response, immune regulation, cell apoptosis, and other pathways. The signaling pathways mainly focus on three aspects: (1) Antivirus. Among the top 39 signaling pathways, 12 are related to antiviral, such as Coronavirus disease—COVID-19. (2) Regulation of the immune system. There are six pathways associated with immune regulation. The overactivation of the immune system leads to a cytokine storm, an essential step in transforming from mild to severe diseases. The four prescriptions may play a role in modulating immunity, avoiding or mitigating cytokine storms by acting on the T cell receptor signaling pathway, Toll-like receptor signaling pathway, NOD-like receptor signaling pathway, Th1, and Th2 cell differentiation, Chemokine signaling pathway, and Fc epsilon RI signaling pathway by interfering with a range of pathophysiological processes, including the recruitment, activation, and secretion of immune cells. (3) Inflammatory response. There are four signaling pathways involved in inflammation, including the IL-17 signaling pathway, Th17 cell differentiation, C-type lectin receptor signaling pathway, and TNF signaling pathway. To sum up, the four CPMs mainly treat COVID-19 through anti-virus, immune regulation, and anti-inflammatory. Twenty-two of the top 39 signaling pathways can be attributed to these three areas, as shown in Table 5. The specific mechanisms of action of the four CPMs on the Coronavirus disease—COVID-19 pathway are shown in Figure 12.

## Conclusion

TCM is the strength and highlight of China’s COVID-19 prevention and control plan. In the fight against COVID-19, TCM has been involved in the prevention, treatment, and rehabilitation. TCM and Western medicine complement and cooperate, which makes an essential contribution to the comprehensive control of COVID-19. In this study, the possible common mechanism of the action of four classic CPMs in treating COVID-19 has been unearthed. Consequently, 101 common active ingredients and 233 common targets were screened out. There were 51 potential targets and 92 related pathways associated with COVID-19. Further analysis revealed that the active ingredients might be flavonoids, such as quercetin, luteolin, and acacetin. The enrichment analysis of 51 potential targets indicated that these targets could be applied to treat COVID-19 by activating broad spectrum antiviral, immune regulatory, and inflammatory responses through the regulation of the Coronavirus disease—COVID-19, cytokine-cytokine receptor interaction, IL-17 signaling pathway, and others. Due to intra-laboratory and multiple conditions limitations, this study explored the common mechanism of action of four PCMs for treating COVID-19 through network pharmacology and molecular dynamics with some limitations. In the future, we will further validate the candidate targets and potential signaling pathways predicted in this study by *in vivo* and *in vitro* experiments. China is willing to unite people from all countries to fight the epidemic and share the experience and achievements of Chinese medicine.

## Data availability statement

The datasets presented in this study can be found in online repositories. The names of the repository/repositories and accession number(s) can be found in the article/Supplementary material.

## Author contributions

HY directed the research and revised the manuscript. LW and ZW performed the research and wrote the manuscript. XW modified the tables and figures and revised the manuscript. LY, JW, and YL revised the manuscript. All authors have read and approved the manuscript.

## Footnotes

1 http://tcmspw.com/tcmsp.php, Ver. 2.3.

2
http://119.3.41.228:8000/tcmid/

3
http://www.talete.mi.it/products/dragon_description.htm

4
https://go.drugbank.com/

5
https://www.uniprot.org/

6
http://genoweb.toulouse.inra.fr:8091/app/example.html

7
https://www.genecards.org/

8
https://www.ncbi.nlm.nih.gov/

9 http://string-db.org/, ver. 11.0.

10
www.cytoscape.org/

11
http://apps.cytoscape.org/apps/cytohubba
