# Supplementary material for: Potential common mechanism of four Chinese patent medicines recommended by diagnosis and treatment protocol for COVID-19 in medical observation period
Source: Front Med (Lausanne). 2022 Oct 26;9:874611. doi: 10.3389/fmed.2022.874611 (PMC9643314; doi:10.3389/fmed.2022.874611)
Supplement: Supplementary file 1 [file Data_Sheet_1.pdf]

1. em.mdp：

```
; em.mdp - used as input into grompp to generate em.tpr
; Parameters describing what to do, when to stop and what to save
integrator    = steep               ; Algorithm (steep = steepest descent minimization)
emtol             =  1000.0               ;  Stop  minimization  when  the  maximum  force  <  1000.0 
kJ/mol/nm
emstep          = 0.01                  ; Minimization step size
nsteps          = 50000                 ; Maximum number of (minimization) steps to perform
; Parameters describing how to find the neighbors of each atom and how to calculate the 
interactions
nstlist               = 1               ; Frequency to update the neighbor list and long range forces
cutoff-scheme     = Verlet       ; Buffered neighbor searching
ns_type               = grid          ; Method to determine neighbor list (simple, grid)
coulombtype        = PME            ; Treatment of long range electrostatic interactions
rcoulomb             = 1.0            ; Short-range electrostatic cut-off
rvdw                   = 1.0            ; Short-range Van der Waals cut-off
pbc                     = xyz           ; Periodic Boundary Conditions in all 3 dimensions
```

2. ions.mdp：

```
; ions.mdp - used as input into grompp to generate ions.tpr
; Parameters describing what to do, when to stop and what to save
integrator    = steep               ; Algorithm (steep = steepest descent minimization)
emtol             =  1000.0               ;  Stop  minimization  when  the  maximum  force  <  1000.0 
kJ/mol/nm
emstep          = 0.01                  ; Minimization step size
nsteps          = 50000                 ; Maximum number of (minimization) steps to perform
; Parameters describing how to find the neighbors of each atom and how to calculate the 
interactions
nstlist               = 1               ; Frequency to update the neighbor list and long range forces
cutoff-scheme     = Verlet       ; Buffered neighbor searching
ns_type               = grid          ; Method to determine neighbor list (simple, grid)
coulombtype        = cutoff       ; Treatment of long range electrostatic interactions
rcoulomb             = 1.0            ; Short-range electrostatic cut-off
rvdw                   = 1.0            ; Short-range Van der Waals cut-off
pbc                     = xyz           ; Periodic Boundary Conditions in all 3 dimensions
```

3. nvt.mdp：

```
title                            = Protein-ligand complex NVT equilibration
define                             = -DPOSRES    ; position restrain the protein and ligand
; Run parameters
integrator                     = md              ; leap-frog integrator
nsteps                           = 50000         ; 2 * 50000 = 100 ps
dt                                 = 0.002         ; 2 fs
; Output control
```

```
nstenergy               = 500     ; save energies every 1.0 ps
nstlog                  = 500     ; update log file every 1.0 ps
nstxout-compressed      = 500     ; save coordinates every 1.0 ps
nstvout                 = 500     ; save velocities every 1.0 ps
; Bond parameters
continuation            = no      ; first dynamics run
constraint_algorithm    = lincs   ; holonomic constraints
constraints             = h-bonds ; bonds to H are constrained
lincs_iter              = 1       ; accuracy of LINCS
lincs_order             = 4       ; also related to accuracy
; Neighbor searching and vdW
cutoff-scheme           = Verlet  ; Buffered neighbor searching
ns_type                 = grid    ; search neighboring grid cells
nstlist                 = 20      ; largely irrelevant with Verlet
rlist                   = 1.2
vdwtype                 = cutoff
vdw-modifier            = force-switch
rvdw-switch             = 1.0
rvdw                    = 1.2     ; short-range van der Waals cutoff (in nm)
; Electrostatics
coulombtype             = PME     ; Particle Mesh Ewald for long-range electrostatics
rcoulomb                = 1.2     ; short-range electrostatic cutoff (in nm)
pme_order               = 4       ; cubic interpolation
fourierspacing          = 0.16    ; grid spacing for FFT
; Temperature coupling
tcoupl                  = V-rescale                 ; modified Berendsen thermostat
tc-grps                 = Protein_LIG Water_and_ions ; two coupling groups - more accurate
tau_t                   = 0.1     0.1               ; time constant, in ps
ref_t                   = 310     310               ; reference temperature, one for each group, in K
comm-grps = Protein_LIG Water_and_ions
comm-mode = Angular
; Pressure coupling
pcoupl                  = no      ; no pressure coupling in NVT
; Periodic boundary conditions
pbc                     = xyz     ; 3-D PBC
; Dispersion correction is not used for proteins with the C36 additive FF
DispCorr                = no
; Velocity generation
gen_vel                 = yes     ; assign velocities from Maxwell distribution
gen_temp                = 310     ; temperature for Maxwell distribution
gen_seed                = -1      ; generate a random seed
disre    = Ensemble
```

disre_weighting = conservative
disre_fc = 10000

4. npt.mdp

```
title                    = Protein-ligand complex NPT equilibration
define                   = -DPOSRES   ; position restrain the protein and ligand
; Run parameters
integrator               = md          ; leap-frog integrator
nsteps                   = 50000       ; 2 * 50000 = 100 ps
dt                       = 0.002       ; 2 fs
; Output control
nstenergy                = 500         ; save energies every 1.0 ps
nstlog                   = 500         ; update log file every 1.0 ps
nstxout-compressed       = 500         ; save coordinates every 1.0 ps
; Bond parameters
continuation             = yes         ; continuing from NVT
constraint_algorithm     = lincs       ; holonomic constraints
constraints              = h-bonds     ; bonds to H are constrained
lincs_iter               = 1           ; accuracy of LINCS
lincs_order              = 4           ; also related to accuracy
; Neighbor searching and vdW
cutoff-scheme            = Verlet
ns_type                  = grid        ; search neighboring grid cells
nstlist                  = 20          ; largely irrelevant with Verlet
rlist                    = 1.2
vdwtype                  = cutoff
vdw-modifier             = force-switch
rvdw-switch              = 1.0
rvdw                     = 1.2         ; short-range van der Waals cutoff (in nm)
; Electrostatics
coulombtype              = PME         ; Particle Mesh Ewald for long-range electrostatics
rcoulomb                 = 1.2
pme_order                = 4           ; cubic interpolation
fourierspacing           = 0.16        ; grid spacing for FFT
; Temperature coupling
tcoupl                   = V-rescale                  ; modified Berendsen thermostat
tc-grps                  = Protein_LIG Water_and_ions ; two coupling groups - more
accurate
tau_t                    = 0.1     0.1                ; time constant, in ps
ref_t                    = 310     310                ; reference temperature, one for
each group, in K
; Pressure coupling
pcoupl                   = Berendsen                  ; pressure coupling is on for
NPT
```

```
pcoupltype              = isotropic                 ; uniform scaling of box vectors
tau_p                   = 2.0                       ; time constant, in ps
ref_p                   = 1.0                       ; reference pressure, in bar
compressibility         = 4.5e-5                    ; isothermal compressibility of water, bar^-1
refcoord_scaling        = com
comm-grps = Protein_LIG Water_and_ions
comm-mode = Angular
; Periodic boundary conditions
pbc                     = xyz       ; 3-D PBC
; Dispersion correction is not used for proteins with the C36 additive FF
DispCorr                = no
; Velocity generation
gen_vel                 = no        ; velocity generation off after NVT
disre    = Ensemble
disre_weighting =    conservative
disre_fc = 10000
```

5. md.mdp

```
title                   = Protein-ligand complex MD simulation
; Run parameters
integrator              = md            ; leap-frog integrator
nsteps                  = 25000000      ; 2 * 5000000 = 100000 ps (100 ns)
dt                      = 0.002         ; 2 fs
; Output control
nstenergy               = 5000          ; save energies every 10.0 ps
nstlog                  = 5000          ; update log file every 10.0 ps
nstxout-compressed      = 5000          ; save coordinates every 10.0 ps
compressed-x-grps       = System        ; save the whole system
; Bond parameters
continuation            = yes           ; continuing from NPT
constraint_algorithm    = lincs         ; holonomic constraints
constraints             = h-bonds       ; bonds to H are constrained
lincs_iter              = 1             ; accuracy of LINCS
lincs_order             = 4             ; also related to accuracy
; Neighbor searching and vdW
cutoff-scheme           = Verlet
ns_type                 = grid          ; search neighboring grid cells
nstlist                 = 20            ; largely irrelevant with Verlet
rlist                   = 1.2
vdwtype                 = cutoff
vdw-modifier            = force-switch
rvdw-switch             = 1.0
rvdw                    = 1.2           ; short-range van der Waals cutoff (in nm)
```

```
; Electrostatics
coulombtype              = PME          ; Particle Mesh Ewald for long-range electrostatics
rcoulomb                 = 1.2
pme_order                = 4            ; cubic interpolation
fourierspacing           = 0.16         ; grid spacing for FFT
; Temperature coupling
tcoupl                   = V-rescale                        ; modified Berendsen thermostat
tc-grps                  = Protein_LIG Water_and_ions       ; two coupling groups - more accurate
tau_t                    = 0.1     0.1                      ; time constant, in ps
ref_t                    = 310     310                      ; reference temperature, one for each group, in K
; Pressure coupling
pcoupl                   = Parrinello-Rahman                ; pressure coupling is on for NPT
pcoupltype               = isotropic                        ; uniform scaling of box vectors
tau_p                    = 2.0                              ; time constant, in ps
ref_p                    = 1.0                              ; reference pressure, in bar
compressibility          = 4.5e-5                           ; isothermal compressibility of water, bar^-1
; Periodic boundary conditions
pbc                      = xyz          ; 3-D PBC
; Dispersion correction is not used for proteins with the C36 additive FF
DispCorr                 = no
; Velocity generation
gen_vel                  = no           ; continuing from NPT equilibration
comm-grps = Protein_LIG Water_and_ions
comm-mode = Angular
disre    = Ensemble
disre_weighting =    conservative
disre_fc = 10000
```
